# Protocol: examining the effectiveness of an adaptive implementation intervention to improve uptake of the VA suicide risk identification strategy: a sequential multiple assignment randomized trial

**DOI:** 10.1186/s13012-020-01019-6

**Published:** 2020-07-22

**Authors:** Nazanin H. Bahraini, Bridget B. Matarazzo, Catherine N. Barry, Edward P. Post, Jeri E. Forster, Katherine M. Dollar, Steven K. Dobscha, Lisa A. Brenner

**Affiliations:** 1 VA Rocky Mountain Mental Illness Research, Education and Clinical Center (MIRECC), Rocky Mountain Regional VA Medical Center, 1700 N Wheeling St, Aurora, CO 80045 USA; 2 Department of Psychiatry, University of Colorado Anschutz School of Medicine, Aurora, CO USA; 3 Department of Physical Medicine and Rehabilitation, University of Colorado Anschutz School of Medicine, Aurora, CO USA; 4 VA Program Evaluation and Resource Center (PERC), Palo Alto, CA USA; 5 Ann Arbor VA Health Care System, Ann Arbor, MI USA; 6 Department of Internal Medicine, University of Michigan Medical School, Ann Arbor, MI USA; 7 VA Center for Integrated Healthcare, Syracuse, NY USA; 8 VA Center to Improve Veteran Involvement in Care, Portland VA Health Care System, Portland, OR USA; 9 Department of Psychiatry, Oregon Health & Science University School of Medicine, Portland, OR USA; 10 Department of Neurology, University of Colorado Anschutz School of Medicine, Aurora, CO USA

## Abstract

**Background:**

In 2018, the Veterans Health Administration (VHA) mandated implementation of a national suicide risk identification strategy (Risk ID). The goal of Risk ID is to improve the detection and management of suicide risk by standardizing suicide risk screening and evaluation enterprise-wide. In order to ensure continuous quality improvement (QI), ongoing evaluation and targeted interventions to improve implementation of Risk ID are needed. Moreover, given that facilities will vary with respect to implementation needs and barriers, the dose and type of intervention needed may vary across facilities. Thus, the objective of this study is to examine the effectiveness of an adaptive implementation strategy to improve the uptake of suicide risk screening and evaluation in VHA ambulatory care settings. In addition, this study will examine specific factors that may impact the uptake of suicide risk screening and evaluation and the adoption of different implementation strategies. This protocol describes the stepped implementation approach and proposed evaluation plan.

**Methods:**

Using a sequential multiple assignment randomized trial (SMART) design, two evidence-based implementation strategies will be evaluated: (1) audit and feedback (A&F); (2) A&F plus external facilitation (A&F + EF). Implementation outcomes of interest include uptake of secondary suicide risk screening and uptake of comprehensive suicide risk evaluation (stages 2 and 3 of Risk ID). Secondary outcomes include rates of other clinical outcomes (i.e., safety planning) and organizational factors that may impact Risk ID implementation (i.e., leadership climate and leadership support).

**Discussion:**

This national QI study will use a SMART design to evaluate whether an adaptive implementation strategy is effective in improving uptake of a mandated VHA-wide suicide risk screening and evaluation initiative. If this study finds that the proposed stepped implementation strategy is effective at increasing uptake and maintaining performance improvements, this approach may be used as an overarching QI strategy for other national suicide prevention programs.

**Trial registration:**

ClinicalTrials.gov NCT04243330. Registered 28 January 2020

Contribution to the literature

- This is the first QI project to apply a SMART design to improve implementation of a nationally mandated suicide prevention initiative in the Veterans Health Administration (VHA).
- Creating an adaptive implementation strategy that provides different degrees of implementation support in a step-wise fashion is expected to be an efficient way of improving uptake of suicide risk screening and evaluation in VHA.
- This study will also contribute to a better understanding of organizational factors that impact adoption of different implementation strategies (e.g., technical assistance, audit and feedback, external facilitation) for improving uptake of evidence-based suicide prevention practices.

## Background

In the last decade, the Department of Veterans Affairs (VA) has made significant strides in suicide prevention, particularly for veterans receiving Veterans Health Administration (VHA) care. However, most of these efforts have focused on downstream interventions to reduce suicidal behavior among those already identified to be at high risk. In contrast, more upstream efforts, such as population-based suicide risk screening, have not been systematically implemented across VHA settings. Instead, suicide risk screening and evaluation have traditionally been limited to select patient cohorts or treatment settings (e.g., those with a known psychiatric disorder). However, emerging evidence indicates that many individuals who die by suicide are not identified as having psychiatric disorders and often present for nonbehavioral health care prior to their death [1–3].

**Fig. 1:**
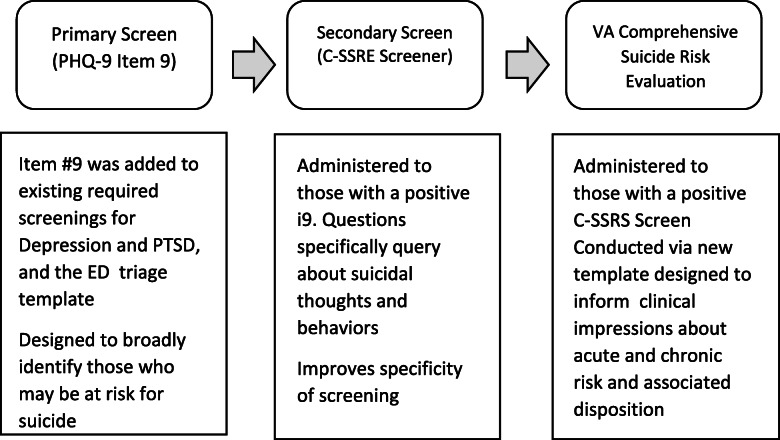
Stages of VA Risk ID for veterans eligible for annual depression and as required PTSD screening

Given that early and accurate detection of suicide risk among all veterans presenting for VHA care is a critical component of *VA’s National Strategy for Preventing Veteran Suicide 2018–2028* [4], VHA leadership mandated implementation of a national suicide risk identification strategy (Risk ID), beginning October 1, 2018 [5]. The goal of Risk ID is to improve the detection and management of suicide risk by standardizing suicide risk screening and evaluation enterprise-wide. Risk ID incorporates staged evidence-informed tools and processes. The three stages include two levels of screening, followed by a comprehensive suicide risk evaluation (CSRE) (Fig. 1).

Risk ID is the largest implementation of population-based suicide risk screening and evaluation in any US healthcare system to date*.* Given the considerable scope of this initiative, several strategies have been employed to support national implementation: informatics tools, educational webinars, facility champions, technical assistance, and clinical performance measures to monitor implementation of Risk ID to fidelity. Despite these efforts, some facilities will face challenges to implementation. In order to ensure continuous quality improvement (CQI), ongoing evaluation and interventions to improve implementation of Risk ID are needed.

A critical element of any CQI program is the ability to reliably measure performance. In preparing for Risk ID implementation, clinical performance measures for patients eligible for screening in ambulatory care settings were developed (e.g., sui2 [timely receipt of secondary suicide risk screen for those with a positive primary suicide risk screen]). These measures provide a standardized way of monitoring implementation of Risk ID across facilities and serve as the basis for CQI interventions, such as audit and feedback (A&F). A&F is defined as “any summary of clinical performance of health care over a specified period of time aimed at providing information to health professionals to allow them to assess and adjust their performance” [6]. A&F trials have shown small to moderate yet worthwhile improvements in performance, and some studies have demonstrated large effect sizes [6]. Recent studies [7] suggest that conceptualizing A&F within a theoretical framework may help improve the effectiveness of A&F interventions. For example, the model of actionable feedback [8], which is rooted in Feedback Intervention Theory (FIT) [9], posits that three cues (timeliness, individualization, and non-punitiveness) presented in hierarchical order are necessary prerequisites to effective feedback and provide increased meaning to make the feedback more actionable. Research has shown that the effects of A&F are maximized when initial performance is low and feedback is non-punitive, provided frequently, and includes specific targets and suggested actions [6, 10], providing empirical support for the actionable feedback model [8–10].

Using A&F as part of a multi-faceted implementation strategy may also enhance its impact.

Ivers and colleagues [11] highlighted the potential of combining A&F with coaching and implementation facilitation (IF) to help providers move from reactions to their data towards planning for change. IF is “a multi-faceted process of enabling and supporting individuals, groups and organizations in their efforts to adopt and incorporate clinical innovations into routine practices” [12]. This can include problem solving and support that occurs in the context of a recognized need for improvement, or it can address a range of implementation challenges through other implementation strategies [12]. Like A&F, IF is likely to be more impactful when its application is driven by an implementation framework (e.g., integrated Promoting Action on Research Implementation in Health Services [i-PARIHS] framework [13]). Such models can help guide thinking about how to apply IF to a particular implementation effort [13] and how to include distinct strategies (e.g., A&F) as part of IF to target specific implementation barriers.

Consistent with the i-PARIHS framework [13], successful implementation of Risk ID (the *innovation*) depends on changing multiple behaviors of multiple types of people (e.g., health professionals, managers, administrators—the *recipients*) in the complex *context* of a busy medical center. Behavior change is complicated, which is why multifaceted approaches that incorporate a variety of implementation strategies are needed to address the range of barriers that can impact implementation. More intensive strategies, such as facilitation, could certainly enhance the effectiveness of A&F; however, it also increases the cost of the intervention. Thus, consideration of when and for which facilities certain implementation strategies should be provided is necessary. Some facilities (i.e., early adopters) may not need additional intervention. Even among facilities that require additional intervention, the dose and type of intervention needed may vary. Therefore, creating an *adaptive implementation strategy* that provides different degrees of implementation support in a step-wise fashion is expected to be an efficient way of improving uptake of Risk ID. Drawing on both the actionable feedback model [8] and i-PARIHS framework [13], we will be testing whether a staged implementation approach consisting of A&F followed by augmentation with external facilitation (EF) improves uptake of Risk ID for facilities that continue to demonstrate low uptake with A&F alone. The rationale for starting with A&F as a first line intervention is that it is a relatively low-intensity/low-cost strategy. Because the A&F intervention will be based on data extracted from medical record, no additional data collection is necessary making it more feasible to implement on a larger scale. EF, on the other hand, requires more resources. Thus, beginning with a less resource-intensive intervention and augmenting with a more targeted, resource-intensive intervention to address specific barriers among sites that continue to perform below expectations may be a more cost-effective approach to improving implementation of Risk ID.

### Study aims

This study is part of a larger national QI project (i.e., partnered evaluation) funded by VA Quality Enhancement Research Initiative (QUERI) and the Office of Mental Health and Suicide Prevention (OMHSP). The QI aims of this project are to evaluate the uptake of Risk ID across VHA facilities and to provide stepped implementation support to facilities that are not meeting a benchmark determined by OMHSP. This project will gather additional information from VHA employees to examine specific factors that may impact the uptake of Risk ID and the adoption of different implementation strategies. This information is intended to contribute to generalizable knowledge regarding organizational factors (e.g., organizational climate, leadership support) that can influence the implementation of evidence-based suicide prevention practices in general medical settings.

#### Primary aim

Among sites that do not meet the performance benchmark following implementation as usual (IAU), does the addition of A&F significantly improve implementation of secondary screening and CSRE compared to IAU alone?

#### Secondary aim 1

Among sites that continue to not meet the performance benchmark after A&F, does the addition of EF significantly improve implementation of secondary screening and CSRE compared to A&F alone?

#### Secondary aim 2

Among sites that meet the performance benchmark following A&F alone, is performance maintained following discontinuation of A&F?

#### Exploratory aims

We will evaluate the clinical impact of Risk ID by examining whether veterans who receive the CSRE (stage 3) are more likely to receive a safety plan than veterans who screen positive on the primary screen only (stage 1).

#### Additional research aims

We will also examine contextual factors (e.g., leadership support, organizational climate) that may impact the (a) implementation of Risk ID and (b) adoption of the implementation interventions.

## Methods

This is a QI study to improve the implementation of a nationally mandated suicide risk screening and evaluation program (Risk ID) in ambulatory care settings. At the time of protocol submission, the trial intervention had already started and collection of outcomes for QI portion had begun. This study was reviewed and approved by the local VA Research and Development Committee, and the additional research aims were reviewed and approved by local IRB and VA Research and Development Committee. This study was registered as a clinical trial (ClinicalTrials.gov ID: NCT04243330).

### Evaluation framework and study design

The reach, effectiveness, adoption, implementation, and maintenance qualitative evaluation for systematic translation (RE-AIM QuEST [14]) method will provide an overarching framework for testing the impact of the proposed adaptive implementation strategy and the impact of the clinical innovation (Table 1).

**Table 1:** Application of RE-AIM, level of evaluation, operationalization, and data sources

RE-AIM domain	Operationalization	Data sources	QI vs research	Evaluation time points
**Level of evaluation: clinical innovation**				
**Reach**	The absolute number and representativeness of veterans that received the primary, secondary screens, and CSRE.	Administrative (CDW) data	QI	Follow-up 2
**Effectiveness**	Whether veterans who receive the CSRE are more likely to receive a safety plan (SP) compared to veterans who screen positive on the primary screen only.	Administrative (CDW) data	QI	Follow-up 2
**Implementation**	Percentage of eligible veterans sampled at each facility who receive the different stages of VA Risk ID as intended; barriers and facilitators to implementation to fidelity.	Administrative (CDW) data; key informant (KI) and debriefing interviews; surveys	QI and research	Baseline, follow-up 1, follow-up 2
**Level of evaluation: implementation strategy**				
**Effectiveness**	Effect of A&F intervention on implementation of VA Risk ID to fidelity compared to IAU alone. Effect of A&F + EF intervention on implementation of VA Risk ID to fidelity compared to A&F alone.	Administrative (CDW) data (primary), EPRP measures (secondary)	QI	Baseline, follow-up 1, follow-up 2
**Adoption**	Number of sites randomized to the implementation interventions that participated. Characteristics of participating/non-participating sites and reasons for participating/not participating.	Key informant (KI) and debriefing interviews	QI and research	Follow-up 1, follow-up 2
**Implementation**	Percent of sampled instances of implementation intervention delivered to fidelity (i.e., met criteria for adherence).	Fidelity checklists; KI and debriefing interviews	QI	Baseline, follow-up 1, follow-up 2
**Maintenance**	Maintenance of adequate implementation following removal of A&F; sustained implementation of VA Risk ID for high performers	Administrative (CDW) data (primary); EPRP measures (secondary)	QI	Follow-up 1, follow-up 2

**Fig. 2:**
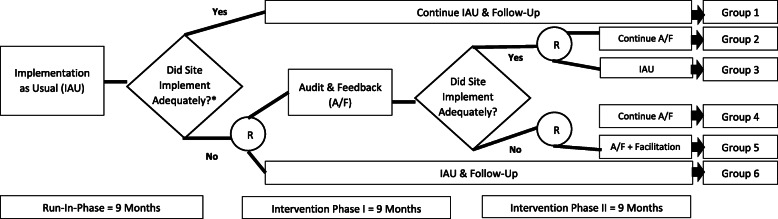
SMART design and intervention phases. Asterisk indicates adequate implementation = completion of secondary screening and CSRE for 80% or more of eligible patients (Note: this benchmark is subject to change based on program office’s determination); R, randomization

The primary and secondary aims (i.e., effectiveness and maintenance of the implementation strategy) will be evaluated using a sequential multiple assignment randomized trial (SMART) design [15]. We will employ a mixed-methods approach to evaluate additional research aims. This project will occur over three phases: run-in phase, intervention phase I, and intervention phase II (Fig. 2).

### Site selection

Up to 140 Veterans Affairs Medical Centers (VAMCs) across the country will be enrolled in the SMART. Sites will be allocated to interventions based on performance (i.e., pre-determined benchmarks for adequate implementation). Although no patients will be recruited for this project, patient level data from electronic medical records (EMR) will be used for outcomes.

### VHA employee recruitment

VHA providers and leadership involved in implementation of Risk ID will be invited to participate in key informant interviews and surveys at three different time points during the larger QI study (Table 1). For the interviews and surveys, we will recruit up to 50 and 150 employees (e.g., primary care [PC] and mental health [MH] leadership and providers, quality managers), respectively, across 20 sites that differ based on geographical location, facility complexity level, and performance level.

### Site randomization

The primary tailoring variable (PTV) determines the set of randomized intervention options. In this SMART, the PTV will be based on Columbia–Suicide Severity Rating Scale (C-SSRS) Screener fallouts and CSRE fallouts. The cutoff for both must be 80% or higher (i.e., less than 20% fallouts) to be considered adequate implementation. This cutoff was chosen with input from the program office. This benchmark is meant to represent a performance target that all facilities are expected to work towards. The program office may adjust this benchmark based on additional data gathered.

The unit of intervention is the site, and randomization will occur at the site/facility level. Facilities that are not meeting the performance benchmark at baseline month 9 will be randomized 1:1 (R1) to receive A&F or continued IAU for 9 months. A&F sites that do not meet the performance benchmark at the 9th interventional month will be randomized 1:1 (R2) to either continue A&F or augmentation with EF (A&F + EF) for an additional 9 months. A&F sites that meet the performance benchmark will be randomized 1:1 (R3) to either continue A&F or discontinue A&F and return to IAU. R1 will be stratified by facility complexity level and level of performance at baseline. The Facility Complexity Model [16] classifies VA medical facilities at levels 1a, 1b, 1c, 2, or 3. Levels 1a–1c will be considered high complexity (*N* = 85; 61%), level 2—medium complexity (*N* = 24; 17%), and level 3—low complexity (*N* = 31; 22%). Higher complexity facilities serve a greater number of patients and offer a more comprehensive range of services. Additionally, the average of the C-SSRS Screener and CSRE fallout rates for the 9th baseline month will be calculated for each facility to be randomized (R1), and the median of these averages will be used to determine stratification by performance level (i.e., low—above median fallout rate or moderate—below median fallout rate). R2 will also be stratified by facility complexity level and performance level (based on the median fallout rate at the 9th month of phase 1). R3 will be stratified only by facility complexity level as all sites will have met the benchmark. These procedures will ensure that intervention groups are balanced for site variables that may correlate highly with outcomes. If less than 12 sites are randomized at R2, stratification will only occur based on facility complexity. If all facilities are at the same complexity level, only median performance level will be used for stratification.

### Implementation strategies

A summary of the implementation conditions and intervention components are described in Table 2.

**Table 2:** Implementation conditions and intervention components

Intervention component	Component description	Intervention condition		
		Implementation as usual (IAU)	Audit and feedback (A&F)	Audit and feedback plus external facilitation (A&F + EF)
**Facility champion**	An identified person at the facility who supports implementation at their site by accessing and disseminating available resources.	X	X	X
**Risk ID SharePoint site**	A website which includes information and resources to support implementation.	X	X	X
**Webinar series**	Risk ID overview and practice trainings.	X	X	X
**Technical support email**	Designated email group to respond to implementation questions from the field.	X	X	X
**Technical assistance call**	A weekly video conference call designed to share updates, materials, and answer questions from the field.	X	X	X
**Fallout report**	A report identifying patients who did not receive the indicated level(s) of screening and/or evaluation.	X	X	X
**Monthly adherence report**	Facility level report provided to leadership each month that summarizes adherence for secondary suicide screening and CSRE.	X	X	X
**Risk ID performance dashboard**	An interactive dashboard that reports facility adherence to Risk ID requirements on an ongoing basis. This includes the ability to compare metrics with national, VISN, and similar-sized facilities; performance across time; and breakdown of performance across clinics/divisions within a parent facility.		X	X
**Risk ID performance dashboard toolkit**	Guidance documents and videos on how to use the Risk ID dashboard to identify areas for improvement.		X	X
**Actionable feedback**	Tailored recommendations for performance improvement.		X	X
**External facilitation**	In person or virtual support to include stakeholder engagement, education, collaborative problem solving and goal setting, increased direct communication, and ongoing support.			X

#### Implementation as usual

Consistent with the evidence-based system for innovation support logic model [17], we have combined tools, training, and technical assistance (TA) with a quality assurance measure to develop a robust support system for implementation. IAU was made available to all VHA facilities starting in July 2018. *Proactive TA* is delivered via weekly conference calls and a support email address. These resources facilitate proactive, rather than reactive, problem-solving with the field [18, 19]. When indicated, we also offer phone calls with individual providers or teams to collaboratively identify flexible implementation solutions that will allow fidelity to be maintained, a common implementation challenge [19]. IAU also includes a SharePoint site which houses a variety of *tools* developed for Risk ID (e.g., guidance documents, checklists). The implementation team also conducted a webinar series, offering *training* on the overall strategy and practice components. These live webinars were converted into recorded trainings in the VA Talent Management System (TMS) so that employees can access them anytime. Finally, a fallout report was developed for *quality assurance*. This report provides information about patients who did not receive indicated levels of the screening and/or evaluation. In addition, the chief mental health officers and primary care leads receive a monthly report detailing the adherence scores for C-SSRS and CSRE uptake for all of the facilities in their Veterans Integrated Service Network (VISN).

#### Audit and feedback (A&F)

The A&F intervention will be guided by the model of actionable feedback [8] and other best practices [10, 11]. The following components will be incorporated into the A&F design and delivery: (i) individualized performance data (site level), (ii) frequent delivery intervals (monthly), (iii) comparisons with other sites, (iv) graphical and text form displays of information, (v) constructive, non-punitive tone, (vi) target performance or benchmark provided, and (vii) specific actions or recommendations for meeting the target. The audit component will include the clinical performance measures for the different Risk ID practices along with more detailed information from the fall out reports. Data will be transformed into different levels of information and presented through an interactive dashboard. In addition, information from the dashboard will be exported monthly and emailed to specific users at each facility (e.g., PC service chief and quality manager). An accompanying toolkit will be developed to guide and educate users on how the information in the dashboard can be used to identify potential areas for process improvement. Various prototypes will be user-tested during the run-in phase, and feedback from potential end users will be obtained to develop a final prototype for use during the phase I intervention.

#### External facilitation

The facilitation approach proposed for this study is grounded in the i-PARIHS framework [13]. The *recipients* of Risk ID (i.e., the *innovation*) include patients, providers, teams, and local leadership. Facilitation will involve gaining an understanding of these recipients’ key characteristics such as: motivation, values and beliefs, goals, skills and knowledge, time, resources and support, and power and authority [13]. Facilitators will also learn about the *inner and outer contexts* of the system in which implementation is occurring. The inner context includes the immediate setting for implementation (e.g., the clinic in which they work) and organization in which the clinic is embedded (e.g., VA medical center). The outer context is the VHA and the related policies, regulatory frameworks, and political environment that impact its functioning [13]. Facilitation will be conducted by a team of facilitators who will employ the facilitation process (a set of strategies and actions) to improve uptake of Risk ID by the recipients. Given that it is unlikely that sites have an internal facilitator who is well-versed in implementation knowledge and skills [12, 20], we will utilize EF. External facilitators will work in collaboration with the facility Risk ID champions. The external facilitator will be trained and mentored by an expert facilitator utilizing a QUERI-supported manual [21] and 2-day in-person or virtual training developed by Dr. Dollar and colleagues [12].

### Outcomes and measures

#### Primary outcomes—C-SSRS screener and CSRE uptake

There will be two primary outcomes for evaluating effectiveness of the implementation interventions: C-SSRS Screener uptake and CSRE uptake. C-SSRS uptake is the percentage of unique individuals in ambulatory care who had a positive primary screen and received the C-SSRS screener as intended (same day and by the appropriate provider). CSRE is the percentage of unique individuals in ambulatory care who had a positive C-SSRS screener and received the CSRE as intended (same day and by the appropriate provider). Primary data source for C-SSRS screener and CSRE uptake will be the VA Corporate Data Warehouse (CDW). CDW is a database organized into a collection of data domains derived from the VHA electronic health record. It contains records of inpatient and outpatient care including dates and location of care, as well as associated international classification of diseases (ICD) 10 codes.

Secondary data sources for C-SSRS and CSRE uptake will be two clinical performance measures extracted from VA’s EPRP [22] that reflect key practice elements of Risk ID: secondary suicide screening (sui 2) and the CSRE (csra1). These measures are currently in pilot status as they are being validated. EPRP measures are routinely abstracted monthly and represent a smaller number of cases than CDW data. A data use agreement has been obtained to facilitate the use of EPRP data for this QI project.

#### Reach and clinical impact

For exploratory aim 1, specific sources of CDW data will include (1) patient socio-demographic characteristics that allow for comparison between veterans reached and all veterans eligible for screening (2) patient responses and results from the screens for depression, PTSD, and C-SSRS that are administered within mental health assistant; (3) note templates containing the CSRE; and (4) health factors recording responses to the CSRE and date of CSRE administration. These sources of data will also serve as data sources for exploratory aim 3. Additionally, we will gather health factors specific to the suicide prevention safety plan details and dates. Free-text progress notes may also be abstracted if needed.

#### Implementation strategy fidelity

##### Debriefing interviews

Semi-structured interviews will be conducted by an independent evaluator monthly with team members implementing IAU during the three project phases, A&F during intervention phases I and II, and EF during intervention phase II. The purpose of the interviews is to gather information regarding implementation activities, program and implementation modifications, and implementation barriers and facilitators. This information will be used to complete the A&F and EF Fidelity checklists (see below).

##### Activity logs

The IAU activity log contains the components of IAU and will be completed monthly throughout each study phase by the independent evaluator. The EF activity log [12] will be completed by facilitators weekly to track the facilitation activities completed for each site receiving facilitation.

##### Fidelity checklists

Fidelity checklists will be used to ensure that A&F and EF were delivered as intended. The A&F Fidelity checklist will be completed during each month of phases I and II by the independent evaluator. To complete the checklist, the evaluator will review a random sample of 25% of sites receiving A&F. If a best practice was not completed, the evaluator will contact the A&F team to inquire why. At the end of phase II, the evaluator will use information gathered in the debriefing interviews and EF activity log to complete the EF checklist, which is based on core IF activities identified through a scoping review and rigorous consensus process [23].

#### Implementation barriers and facilitators

##### Key informant interviews

Semi-structured interviews will be conducted with PC and MH leadership and providers, facility champions, and quality managers after each of the three project phases via telephone. Interviews will explore barriers/facilitators to Risk ID implementation as well as factors that impact the adoption of the implementation strategies. All interviews will be conducted by the independent evaluator to reduce potential bias. Interviews will be audio recorded, and recordings will be transcribed.

*Implementation Climate Scale (ICS*) [24] is an 18-item questionnaire that assesses the degree to which a facility’s organizational climate is supportive of program integration and implementation. The ICS includes six factors to capture the elements of the organizational environment identified as most vital to the implementation of evidence-based practice (EBP). These factors include selection for openness, recognition for EBP, selection for EBP, focus on EBP, educational support for EBP, and rewards for EBP. Scores range from 0 to 72 with higher scores reflecting better organizational climate for facilitating implementation of an evidence-based practice (i.e., Risk ID). Analyses of construct validity have found the ICS to be strong to moderately correlated to related strategic climate indicators such as the service climate and organizational change [24, 25]. Additionally, ICS was found to have high internal reliability as a measure used by an individual or by a group or team in mental health-related fields [24, 25].

*Implementation Leadership Scale (ILS)* [26] is a 12-item survey that assesses the degree to which a facility’s leadership is supportive and helpful in program integration and implementation. The four sub-scales include productive leadership, knowledgeable leadership, supportive leadership, and perseverant leadership. Scores range from 0 to 48 with higher scores reflecting greater levels of leadership support for implementation of an evidence-based practice (i.e., Risk ID). Reliability and validity assessments have been conducted in acute care settings with frontline nurse managers and in substance abuse disorder treatment organizations. In both studies, the ILS demonstrated high internal constancy reliability and moderate to high convergent and discriminant validity [27, 28].

Interview and survey data will be captured using REDCAP. REDCAP is a secure web application approved by the VA Office of Information & Technology (OI&T) and designed to support data capture for research studies, providing user-friendly web-based case report forms, real-time data entry validation (e.g., for data types and range checks), audit trails, and a de-identified data export mechanism to common statistical packages (e.g., SPSS, SAS).

### Analyses

All data collected from VA employees participating in interviews and surveys will be kept on secure VA servers located behind the VA firewall accessible only to study staff by way of public key infrastructure (PKI) cards and passwords. De-identified electronic data will be available to the study team for data analysis and will be stored on a password-secured network server behind the VA firewall which is only accessible via PKI card and password.

#### Sample size and power

Power was calculated using the two sample *t* test procedure in Power and Sample Size (PASS 16). As there are two primary outcomes, alpha is set to 0.025. The FY2018 first quarter numbers for a similar performance measure (sre1, timely SRE if positive PTSD, or major depressive disorder screen) across 140 facilities was used to obtain an estimated standard deviation for C-SSRS and CSRE uptake of 0.10. Power was calculated for two scenarios given the preliminary fallout data noted above. Assuming proportions of 0.8 and 0.9 not adequately implementing at month 9 of the run-in phase such that *N* = 112 and *N* = 126 are eligible for randomization at R1, this would provide 80% power to detect a difference in change between A&F and IAU of 0.059, and 0.055, respectively. Regarding secondary aim 1, assuming alpha = 0.05, SD = 0.10, and facilities eligible for randomization at R2 of 56 and 63, there is 80% power to detect a difference in change between continued A&F and A&F + EF of 0.076 and 0.072, respectively. Given the same assumptions for secondary aim 2 as used for secondary aim 1, the detectable differences in change are the same for examining continued A&F versus discontinued A&F.

#### Primary aim

Using linear regression, change in C-SSRS screener uptake and CSRE uptake from the 9th month of the baseline period to the 9th month of the first interventional phase will each be modeled as a function of group (A&F vs. IAU), the baseline outcome value, the stratification variables of facility complexity and baseline performance, and geographic region. Inference will be made based on the coefficient associated with the group variable, and 97.5% confidence intervals (CI) will be reported (alpha = 0.025). A similar analysis will be used to determine the effect of A&F on the change in the EPRP outcome for the secondary screeners and CSRE, with 95% CIs reported.

#### Secondary aims

To test the effect of the addition of EF for those who do not implement adequately after receiving A&F, the change in C-SSRS Screener uptake and CSRE uptake from the 9th month of phase 1 (baseline for phase 2) to the 9th month of phase 2 will be modeled as a function of group, the baseline outcome value, the stratification variables, and geographic region (if sample sizes allow for control of this variable). Inference will be made based on the coefficient associated with the group variable, and 95% CIs will be reported. A similar analysis will be used to determine the effect of the addition of EF on the change in uptake for the C-SSRS screener and CSRE, with 95% CIs reported. An analysis similar to that described for secondary aim 1 will be employed to investigate the effect of discontinuing A&F for those who implemented adequately at the end of phase 1. Lastly, we will use a data-driven approach to characterize each of the outcomes over time for each group outlined in Fig. 2 (groups 1–6). Mixed effects models with a random intercept and slope will be used to model each of the outcomes as a function of categorical groups 1–6 and an interaction between group and a B-spline transformation on time (allowing the outcome to vary smoothly over time, using 21-time points, i.e., the 9th baseline (run-in) month and every month of each interventional phase) such that each group will have its own trajectory. The trajectory for each group will be plotted with pointwise confidence intervals

#### Exploratory aims

We will examine whether veterans who receive the CSRE are more likely to receive a safety plan within 2 weeks than those who screen positive on the primary screen and/or negative on the secondary screen only. To account for clustering of veterans within facility, a mixed-effects logistic regression will be used to model the outcome of safety plan within 2 weeks (yes/no) as a function of group (receipt of CSRE/positive on primary screen or negative secondary screen only) with a random subject within facility effect. Additionally, to determine if receipt of a timely safety plan depends on whether veterans are considered to be at low, moderate, or high acute risk of suicide, this model will be repeated with the addition of a group by (categorical) acute risk interaction. Odds ratios for receipt of CSRE relative to positive on primary screen or negative on secondary screen only will be reported for each level of acute risk with 95% confidence intervals.

#### Additional research aims

Key informant interviews and surveys will be used to examine factors influencing adoption of the implementation interventions and barriers and facilitators of implementing VA Risk ID to fidelity. All qualitative data sources, including interview transcripts and documents will be compiled and managed using the Nvivo V. 9.0 software. We will take a general inductive approach. Specifically, data analysis will be determined by both the research objectives (adoption of Risk ID and implementation strategies, organizational climate) and multiple readings and interpretation of the raw data (i.e., content analysis). The goal is to establish clear links between the research objectives and the summary findings derived from the raw data.

Survey data will be scored based on standard scoring instructions. Summary scores for different domains will be calculated and compared across facilities over time. For continuous variables, linear mixed models will be used with a random facility effect and non-normal data will be transformed.

### Trial status

Trial is currently in run-in phase. Site identification and randomization for phase I will occur in July of 2020.

## Discussion

VHA has been a pioneer in the field of suicide prevention over the last decade. Risk ID is further evidence of VA leading suicide prevention in the US, which includes the ability to move upstream to improve earlier detection of suicide risk in all veterans presenting to VHA care. VHA has also been a leader when it comes to continuous quality improvement (CQI) methods to ensure that evidence-based programs, such as Risk ID, can be delivered to fidelity in routine clinical settings and lead to improved patient outcomes. Though the evidence base for CQI programs is growing both within and outside VHA, there remains considerable variability in the studies undertaken with respect to approaches used, populations studied, and outcomes reported [29]. Despite this variability, common elements of successful CQI involves some level of audit and feedback and fostering communication and collaboration among healthcare providers and leadership [29]. The proposed study includes these strategies. Furthermore, it is designed to expand knowledge in the area of healthcare CQI by examining how the delivery, dose and timing of these strategies can be tailored to optimize both implementation and clinical outcomes across VHA facilities. Specifically, the use of a SMART design provides a rigorous way to evaluate a pragmatic and scalable CQI approach in which the level and type of intervention provided is tailored to facility performance over time.

While the use of SMART designs and adaptive strategies in implementation science is emerging, their specific application in CQI has been limited. Currently, a number of ongoing trials are using SMART designs to develop and test adaptive strategies for improving the implementation of evidence-based programs for mood disorders [30] and postpartum depression programs [31]. Contributing to work in this area, this study uses a national VHA mandated suicide prevention program as the basis of a study to examine the impact of different implementation strategies among sites that are not responding to standard implementation support. Though the standard implementation support offered for this program encompasses a wide range of strategies, it is anticipated that several facilities may need additional implementation support to meet Risk ID performance benchmarks.

The selection of A&F as a first line intervention was designed to maximize resources and enhance scalability. Specifically, the A&F tool used in this study will transform relevant data from the EHR into information that is available to users through an interactive dashboard. Different levels of information presented graphically may allow users to filter, drill down, and further explore performance. Furthermore, an accompanying toolkit will help guide and educate users on how to utilize the information presented to identify potential areas for process improvement. The A&F tool is designed to facilitate communication and collaboration between healthcare professionals at the facility level who are involved in the implementation of Risk ID. However, it is important to recognize that various contextual factors, such as organizational climate and leadership support for expanding suicide prevention practices into medical settings may impact the degree to which professionals use this tool to collaboratively trouble-shoot and problem-solve implementation issues. Thus, external facilitation, a more resource intensive intervention specifically designed to foster collaboration, communication, and active problem-solving among healthcare professionals, will be provided to facilities that continue to perform below benchmark even after receiving A&F. Augmenting A&F with external facilitation for select facilities will ensure that resources are directed to facilities that need the additional support.

The mixed-methods design of this study also allows for a better understanding of the conditions under which CQI interventions may be more effective and the characteristics of the healthcare system that may influence effectiveness. For example, this study will examine the impact of organizational factors on both Risk ID uptake and adoption of the implementation strategies. Variations in leadership climate and support across VHA facilities may point to some system-level barriers that can be better targeted through more complex interventions, such as EF. However, these factors may also influence the adoption of these implementation approaches (e.g., willingness to engage in EF). Thus, further examination of contextual factors may help inform whether other system-level interventions are needed to target barriers to implementing suicide prevention practices in certain VHA settings.

Due to the timing of this project, the onset of COVID-19 is another important factor to consider. Changes brought on by COVID-19, such as workforce composition, transition of routine PC and MH appointments to telehealth, and increases in acute stress related to physical distancing, changes in social support, and employment may have different impacts on rates of suicide risk screening and evaluation. Preliminary examination of screening and evaluation rates following COVID-19 suggest that while the overall volume of appointments has decreased, screening is still occurring across VHA facilities. Most importantly, the overall adherence to the different stages of Risk ID is similar to pre-COVID-19 baseline adherence rates. Nonetheless, contextual factors related to COVID-19 that could impact Risk ID will be continuously monitored, and these changes will be accounted for in subsequent analyses as warranted.

## Data Availability

Not applicable

## Abbreviations

A&F: Audit and feedback
AI: Adaptive intervention
CDW: Corporate data warehouse
CI: Confidence intervals
CSRE: Comprehensive suicide risk evaluation
C-SSRS: Columbia–Suicide Severity Rating Scale
EF: External facilitation
EMR: Electronic medical record
EPRP: External Peer Review Program
FIT: Feedback Intervention Theory
IAU: Implementation as usual
ICD: International Classification of Disease
ICS: Implementation climate survey
IF: Implementation facilitation
ILS: Implementation Leadership Scale
i-PARIHS: Integrated Promoting Action on Research Implementation in Health Services
KI: Key informant
OMHSP: Office of Mental Health and Suicide Prevention
PASS: Power and sample size
PC: Primary care
PCMHI: Primary care mental health integration
PTSD: Post traumatic stress disorder
PTV: Primary tailoring variable
QI: Quality improvement
QUERI: Quality Enhancement Research Initiative
RE-AIM QuEST: Reach, Effectiveness, Adoption, Implementation, and Maintenance Qualitative Evaluation for Systematic Translation
Risk ID: Suicide risk identification strategy
SMART: Sequential multiple assignment randomized trial
SP: Safety plan
TA: Technical assistance
TMS: Talent management system
VA: Department of Veterans Affairs
VAMC: Veterans Affairs Medical Center
VHA: Veterans Health Administration
